# Systemic Inflammation in C57BL/6J Mice Receiving Dietary Aluminum Sulfate; Up-Regulation of the Pro-Inflammatory Cytokines IL-6 and TNFα, C-Reactive Protein (CRP) and miRNA-146a in Blood Serum

**DOI:** 10.4172/2161-0460.1000403

**Published:** 2017-11-29

**Authors:** AI Pogue, V Jaber, Y Zhao, WJ Lukiw

**Affiliations:** 1 Alchem Biotech, Toronto ON, Canada; 2 LSU Neuroscience Center, Louisiana State University Health Sciences Center New Orleans, New Orleans LA, USA; 3 Department of Anatomy and Cell Biology, Louisiana State University Health Sciences Center New Orleans, New Orleans LA, USA; 4 Department of Ophthalmology, Louisiana State University Health Sciences Center New Orleans, New Orleans LA, USA; 5 Department of Neurology, Louisiana State University Health Sciences Center New Orleans, New Orleans LA, USA

**Keywords:** C57BL/6J mice, microRNA-9 (miRNA-9), microRNA-125b (miRNA-125b), miRNA-146a (miRNA-146a), aluminum sulfate, C-reactive protein (CRP), interleukin-6 (IL-6), tumor necrosis factor alpha (TNFα), systemic inflammation (SI)

## Abstract

A number of experimental investigations utilizing different murine species have previously reported: (i) that standard mouse-diets supplemented with physiologically realistic amounts of neurotoxic metal salts substantially induce pro-inflammatory signaling in a number of murine tissues; (ii) that these diet-stimulated changes may contribute to a systemic inflammation (SI), a potential precursor to neurodegenerative events in both the central and the peripheral nervous system (CNS, PNS); and (iii) that these events may ultimately contribute to a chronic and progressive inflammatory neurodegeneration, such as that which is observed in Alzheimer’s disease (AD) brain. In these experiments we assayed for markers of SI in the blood serum of C57BL/6J mice after 0, 1, 3 and 5 months of exposure to a standard mouse diet that included aluminum-sulfate in the food and drinking water, compared to age-matched controls receiving magnesium-sulfate or no additions. The data indicate that the SI markers that include the pro-inflammatory cytokines interleukin-6 (IL-6) and tumor necrosis factor alpha (TNFα), the acute phase reactive protein C-reactive protein (CRP) production and a triad of pro-inflammatory microRNAs (miRNA-9, miRNA-125b and miRNA-146a) all increase in the serum after aluminum-sulfate exposure. For the first time these results suggest that *ad libitum* exposure to aluminum-sulfate at physiologically realistic concentrations, as would be found in the human diet over the long term, may predispose to SI and the potential development of chronic, progressive, inflammatory neurodegeneration with downstream pathogenic consequences.

## Introduction

### Systemic inflammation (SI) as a precursor to neurodegenerative disease

Systemic inflammation (SI) (i) represents a chronic and global-state of inflammation in human physiology that affects multiple tissues and organ systems; (ii) is a consequence of the release of pro-inflammatory cytokines from endothelial, mast and other immune-related cells into the bloodstream; and (iii) involves the activation and re-activation of the innate-immune system. In SI, blood-borne pro-inflammatory cytokines such as interleukin-6 (IL-6), tumor necrosis factor alpha (TNFα), acute phase reactive proteins such as C-reactive protein (CRP) and related inflammatory mediators such as novel bioactive docosanoids and fatty acids carry inflammatory signals throughout the systemic vasculature that may cross defective physiological barriers between the PNS and CNS [1–6]. These actions appear to further recruit immune cells in the brain and hence exacerbate neuro-inflammation. Indeed SI has been considered as a potential predecessor to the inflammatory-neuropathology characteristic of Alzheimer’s disease (AD) brain by interfering with immunological processes of the CNS and providing inflammatory mediators to further promote AD-type change [4–7].

Activation of SI, chronic inflammatory signaling and pathological stimulation of the innate-immune system may be the result of external environmental factors, biological or chemical agents. Aluminum is an environmentally abundant physiological toxin and the most abundant metallic neurotoxin in our biosphere. Humans bear a surprisingly large aluminum burden throughout their lives and intake an average of about 10 mg Al/day; this occurs largely via inhalation, and the ingestion of medicine, food and water [8–15]. Moreover, although controversial, aluminum intake has been tentatively linked (i) to both SI and neuroinflammatory signaling up-regulation in AD [4,7,13,14]; (ii) to increased oxidative stress, oxidized fatty acids, isoprostane generation and pro-inflammatory signaling in murine transgenic models of neurodegenerative disease (Tg2576) [15]; (iii) to the induction of pro-inflammatory and pro-apoptotic gene expression patterns in human brain cells in primary culture [11]; (iv) to increased production of pro-inflammatory cytokines such as IL-6 in the immune system by aluminum oxide nanoparticles [16]; (v) to the abundance of the SI biomarker C-reactive protein (CRP) in human brain micro vessel endothelial cells that line the cerebral vasculature [6,17]; and most recently (vi) to increased inflammation in the peripheral nervous system in rats supplemented with aluminum in their diet [18].

These experiments were undertaken to advance our understanding of the potential pathophysiological consequences of aluminum sulfate in the diet of wild type control C57BL/6J mice at 0, 1, 3 and 5 months after feeding these mice with aluminum-sulfate in their daily food and drinking water. C57BL/6J mice at 5 months of age are considered mature adults; control animals received no additions or were fed magnesium sulfate (at the same concentration as aluminum sulfate) to normalize for sulfate intake and were analyzed in parallel. The pro-inflammatory cytokines IL-6, TNFα and CRP were assayed using ELISA, and microRNA (miRNA) array-based analyses were used to quantify the levels of several inducible small non-coding RNA (sncRNAs), including the pro-inflammatory microRNAs (miRNAs) miRNA-9, miRNA-125b and miRNA-146a in blood serum. Here for the first time we report a significant stimulation of SI in C57BL/6J mice fed dietary aluminum at physiologically realistic levels. The results suggest that mammalian diets enriched in aluminum can induce several different classes of SI biomarkers in normally healthy wild type control C57BL/6J mice and direct systemic physiology towards pro-inflammatory signaling that supports degenerative neuropathology. Importantly, the well-studied activation of miRNA-146a signaling is a potent precursor to the establishment of pro-inflammatory gene expression programs in the CNS and PNS with significant downstream, pathogenic consequences [17–20].

## Materials and Methods

### C57BL/6J animals

C57BL/6J mice were purchased from a commercial supplier (http://jaxmice.jax.org/jaxnotes/507/507r.html; Jackson Laboratories, Bar Harbor, MA, USA); typically 9 female animals per test group were randomized and selected to receive either standard rodent chow (meal-ground pellets; Standard Laboratory rodent diet 5001; http://www.labdiet.com/cs/groups/lolweb/@labdiet/documents/web_content/mdrf/mdi4/~edisp/ducm04_028021.pdf; LabDiet St. Louis MO, USA), or a diet enriched in Al sulfate (commonly used as food additive and water purification;http://www.labdiet.com/cs/groups/lolweb/@labdiet/documents/web_content/mdrf/mdi4/~edisp/ducm04_028021.pdf; http://www.generalchemical.com/assets/pdf/Dry_Alum_Food_Grade_PDS.pdf; 100 mg Al/kg diet; LD50 for aluminum sulfate in mice ~980 mg Al/kg; both websites accessed 21 November 2017). Control animals received drinking water containing 0.2 mg/L aluminum (endotoxin-free Ultra-Pure Water, TMS-011 EMD Millipore; Sigma-Aldrich, St Louis MO, USA); see https://www.wqa.org/Portals/0/Technical/Technical%20Fact%20Sheets/2014Aluminum.pdf; http://www.who.int/water_sanitation_health/publications/aluminium/en/; both websites accessed 21 November 2017. In addition aluminum-sulfate treated animals received aluminum-sulfate supplemented water (2.0 mg/L aluminum). The aluminum content of both food and water are consistent and realistic in the consumer environment for daily range of human exposure; both food and water were provided *ad libitum* from birth. C57BL/6J mice stopped weaning and started eating either standard rodent chow or a diet enriched in Al at about 1 month of age (time ‘0’); at 0, 1, 3 and 5 months animals were sacrificed, blood (about ~1 ml per animal) was harvested under RNAse-free clean conditions and were subjected to pro-inflammatory cytokine, acute phase protein and sncRNA (miRNA) analysis. All animal procedures were followed and murine tissues handled in strict accordance with the Institutional Biosafety Committee/Institutional Review Board (IBC/IRB) ethical guidelines at the LSU Health Sciences Center, LA 70112 (IBC#12323; IRB#6774).

### Chemicals and reagents, RNA and protein extraction and quality control

Analytical chemicals and reagents used in these experiments were obtained from standard commercial suppliers and were of the highest grades commercially available; all chemicals and reagents were used in accordance with the manufacturer’s specifications and without any additional purification. Age-matched magnesium-sulfate control and aluminum-sulfate-fed C57BL/6J mice at 0, 1, 3 and 5 months of age were analyzed for IL-6, TNFα and CRP from both aluminum-fed and control animals; total blood serum RNA and protein were isolated using TRIzol reagent as previously described and RNA and protein samples were archived for further downstream analysis. Protein concentrations were determined using a dotMETRIC microassay as previously described (sensitivity 0.3 ng protein/ml; Invitrogen, Carlsbad, CA, USA; Chemicon-Millipore, Billerica, Massachusetts, USA) [18,21,22].

### ELISA analysis for IL-6, TNFalpha (TNFα) and C-reactive protein (CRP)

ELISA analysis for the pro-inflammatory cytokines and reactive factors IL-6, TNFα and CRP were performed using a sandwich ELISA system and a mouse IL-6 ELISA Kit (ab222503; Abcam, Cambridge MA, USA; sensitivity 11.3 pg/ml; range 15.6 pg/ml–1000 pg/ml), a mouse TNF alpha ELISA Kit (ab100747, ab208348; colorimetric; sensitivity 9.1 pg/ml; range 46.88–3000 pg/ml; Abcam), and a mouse CRP ELISA Kit (PTX1) (ab157712; colorimetric, sensitivity 0.159 ug/ml; range 0.78 ug/ml–100 ug/ml; Abcam) according to the manufacturer’s instructions.

### Quality control and small non-coding RNA (sncRNA) analysis

RNA was isolated using TRIzol reagent (for blood serum; a monophasic solution of phenol and guanidine isothiocyanate; Cat. No. 15596026, Invitrogen, Carlsbad, CA, USA) and samples were enriched for small RNAs using spin columns, QIAzol lysis reagent, and RNase-free reagents, buffers, RNAse inhibitors and miRNeasy Mini kits (Cat. No. 217004, Qiagen, Germantown MD, USA). RNA quality was assessed using an Agilent Bioanalyzer 2100 (Lucent Technologies, Murray Hill NJ, USA; Caliper Technologies, Mountain View CA, USA). Typically, 1 μL of total RNA sample was loaded on an RNA chip (6000 Nano Labchip; Caliper Technologies, Mountainview, CA, USA) and analyzed for quality control; RNA integrity values were typically between 8.05 and 8.15; all sncRNA- and/or miRNA-enriched samples were made up to a final concentration of 1ug/uL in UltraPure^™^ DNase/RNase/protease-Free distilled water (Cat No. 10977015, ThermoFisher Scientific, Waltham MA, USA). sncRNA abundance analysis including microRNA (miRNA) abundance analysis for miRNA-9, miRNA-125b, miRNA-146a, miRNA-183 and 5S RNA were quantified using microfluidic sncRNA and miRNA arrays as previously described in detail (LC Sciences, Houston TX, USA) [11,18,21].

### Statistical analysis, data interpretation and integrated bioinformatics analysis

For ELISA and miRNA analysis all statistical procedures were analyzed using (*p*, ANOVA) a two-way factorial analysis of variance using algorithms and procedures in the SAS language (Statistical Analysis Institute, Cary, NC, USA) and as previously described [18,21,22]. In the results *p*-values of less than 0.05 (ANOVA) were considered to be statistically significant. All IL-6, TNFα, CRP and miRNA abundance data were collected and analyzed using Excel 2016 (Office 365) algorithms (Microsoft Corporation, Redmond WA, USA); all figures were generated using Adobe Illustrator CC 2015 and Photoshop CC version14.0 (Adobe Corporation, San Jose CA, USA). Figures were generated using Excel 2008 (Microsoft), Adobe Illustrator CS3 ver 11.0 and Photoshop CS2 ver 9.0.2 (Adobe).

## Results

**Figure 1:**
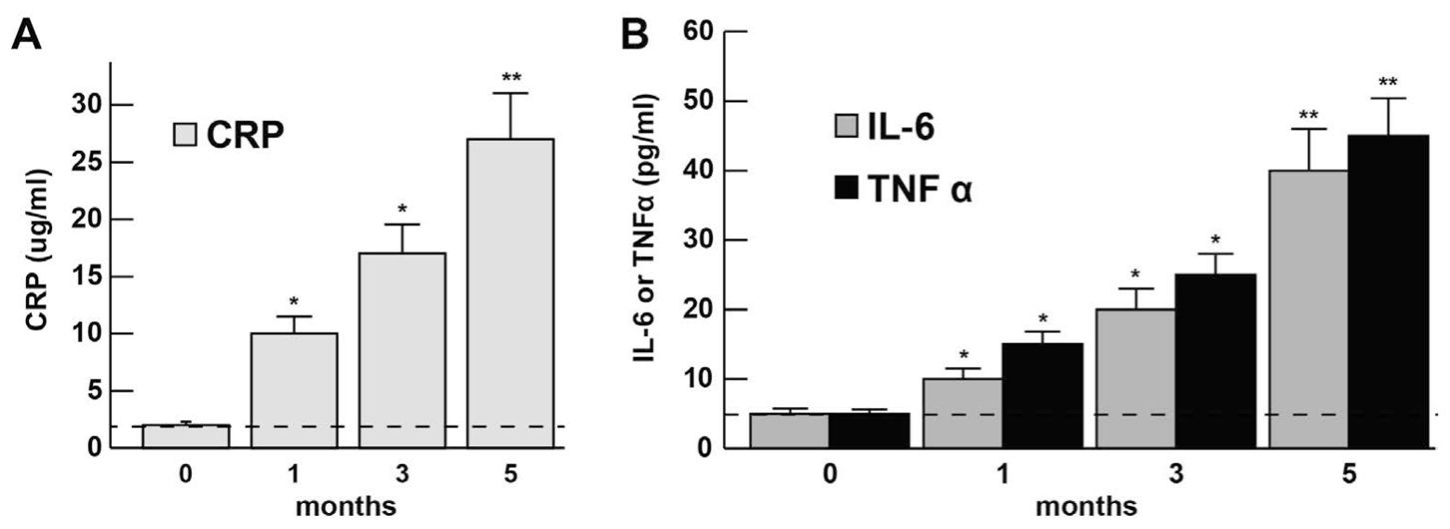
Relative abundance of (A) C-reactive protein (CRP) and (B) the pro-inflammatory cytokines interleukin-6 (IL-6) and tumor necrosis factor alpha (TNFα) in C57BL/6J mouse blood serum after receiving dietary aluminum sulfate in their food and drinking water (*ad libitum*) for 0 (control), 1, 3 and 5 months; as determined by ELISA (see text); N=3 to 5 determinations for each inflammatory marker in each mouse sampled; N=3 mice were analyzed per group; in (A) a dashed horizontal line at 2.0 for CRP; and in (B) a dashed horizontal line at 5.0 (for IL-6 and TNFα) is included for ease of comparison; *p<0.05; **p<0.01 (ANOVA).

The abundance of the CRP, IL-6, and TNFα levels at 0, 1, 3 and 5 months in murine blood serum (approximately blood serum volume per mouse ~1 ml) is shown in bar graph format in (Figures 1A and 1B). CRP, IL-6 and TNFα each showed steady increases over the 0–5 month time period indicating the proliferation of pro-inflammatory markers in blood serum and the potential propagation of a systemic inflammation (SI); at the 1 month time-point CRP, IL-6 and TNFα had increased to ~3.1, 2.1 and 2.7-fold, respectively, over age matched controls; at the 5 month time-point CRP, IL-6 and TNFα were found to increase ~13.1, 8.1 and 9.1-fold over age-matched controls; all 3 inflammatory biomarkers showed very significant increases in the blood serum after 5 months (Figures 1A and 1B). For ease of comparison all CRP levels are shown relative to the CRP levels at ‘0’ time which was ~2.0 ug/ml (Figure 1A); similarly for ease of comparison all IL-6 and TNFα levels are shown relative to the IL-6 and TNFα levels at ‘0’ time which was ~5 pg/ml (Figure 1B). CRP, IL-6 and TNFα all showed progressive increases in abundance from 0 to 5 months. Importantly, there were no significant changes in total body weight between the aluminum-sulfate and ‘no additions’ group’ or magnesium-fed C57BL/6J female mice at any time point over the 0–5 month time course; at 1 month all female mice weighed 10.1 ± 1.7 g (N=36); at 5 months of age control mice weighed 21.2 ± 2.5 g and Al-treated mice weighed 21.7 ± 2.3 g which was not significant; see also for example https://www.jax.org/jax-mice-and-services/strain-data-sheet-pages/body-weight-chart-000664; last accessed 21 November 2017).

**Figure 2:**
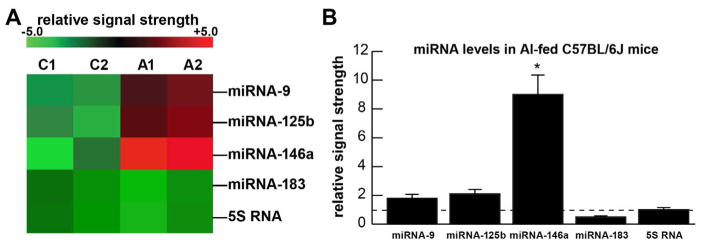
Variable up-regulation of the pro-inflammatory microRNAs miRNA-9, miRNA-125b and miRNA-146a in the blood serum of 5 month old C57BL/6J mice receiving aluminum sulfate in their diet versus controls (both ‘no addition’ and ‘magnesium-sulfate supplemented’ diets gave identical results); (A) miRNA-array generated heat map or ‘cluster diagram’; and (B) bar graph format of the data in (A); in (B) a dashed horizontal line at 1.0 (at the level of 5S RNA) is included for ease of comparison; *p<0.01 (ANOVA).

Figure 2A shows the levels of 3 serum and brain-abundant microRNAs (miRNA-9, miRNA-125b and miRNA-146a) and controls (miRNA-183 and 5S RNA); C1 and C2=blood serum from 2 control C57BL/6J mice receiving magnesium sulfate in their diet; A1 and A2=blood serum from 2 C57BL/6J mice receiving aluminum sulfate in their diet. In the same sample an unchanging control regulatory miRNA (miRNA-183) and an abundant unchanging structural control 5S RNA were included as two sncRNA controls in the same sample; 5S RNA was loaded at one twentieth the concentration of the microRNAs. Figure 2B shows the results of miRNA abundance in bar-graph format of these 3 miRNAs in aluminum-fed over magnesium-sulfate fed controls; both miRNA-9 and miRNA125b showed modest up-regulation to ~1.8 and 2.1-fold over control, respectively, in aluminum sulfate fed animals; the most significantly up-regulated miRNA in these studies was the inflammation and neurodegeneration-associated miRNA-146a to about ~9.1-fold over controls in blood serum after 5 months. Regarding the controls: both ‘no addition’ and ‘magnesium-sulfate supplemented’ diets gave identical results. miRNA increases appear to be the consequence, in part, of an inducible miRNA-9, miRNA-125b and miRNA-146a being up-regulated under conditions of stress as has been observed in both human neuronal-glial (HNG) primary cells and in human brain obtained from short post-mortem interval AD tissue samples [18,21,22].

## Discussion and Conclusion

### Aluminum exposure in humans

Aluminum is the most abundant neurotoxin and genotoxin in our biosphere to which we are constantly exposed over the course of our lives [4,9]. Humans intake an average of about 10 mg Al/day (range 10–1000 mg Al/day) and this occurs mainly via the ingestion of food, water, medicine and inhalation of airborne particulate matter [5–14]. The remarkably low solubility of aluminum at biological pH, and the highly evolved epithelial-, and endothelial-cell-based gastrointestinal (GI) and blood-brain barriers (BBB) prevent this ubiquitous metallotoxin from passage through vascular wall barriers and access to human biological compartments [9,13–16]. Certain physiological molecular carriers and transporters may facilitate aluminum passage through these biological barriers [9]. Ingestion of aluminum from the diet and aluminum that enters the blood stream has been previously shown: (i) to bind with, and be transported by, the serum anion transporters citrate and transferrin in the systemic circulation [9,23–25]; (ii) to selectively associate with micro vessel endothelial cells that line the arteries of the systemic vasculature [26]; (iii) to induce the formation of reactive oxygen and nitrogen species (ROS, RNS), lipid peroxidation in both the central and peripheral CNS [9,26–30]; and (iv) to induce chronic inflammation, systemic oxidative stress, mitochrondrial dysfunction, pro-apoptotic gene expression programs and a progressive inflammatory neurodegeneration in both human brain cells in primary culture and in the CNS and PNS of mammals [6,15,18,27–31].

### Systemic Inflammation, CRP, IL-6, TNFα, miRNA-9, miRNA-125b and miRNA-146a

The purpose of the current research work was to quantify the concentrations and clarify the contributions of CRP, IL-6, TNFα and the levels of the pro-inflammatory microRNAs, including miRNA-9, miRNA-125b and miRNA-146a, in the blood serum of wild-type C57BL/6J mice that were fed aluminum-sulfate in their diet over a time course of 0, 1, 3 and 5 months. The following is a brief discussion of each of these 6 inflammatory mediator-markers quantified to be moderately-to-significantly increased in the systemic circulation of aluminum sulfate-fed animals:

#### C-reactive protein (CRP)

The 224 amino acid (25 kDa; encoded at chr 1q23.2) homopentamer pentraxin, C-reactive protein (CRP), present at an average of between 1.0 and 3.0 ug/mL of blood serum in humans was found to have an average of ~2.0 ug/ml in mouse blood serum at ‘0’ time (Figure 1A). Significantly higher levels of this host innate-immune and inflammatory marker are commonly found in altered physiological states including viral infections, mild inflammation and in the late stages of pregnancy to 10–40 ug/ml, in active inflammation and low grade bacterial infection to 40–200 ug/ml, and in severe burns and in moderate to high grade bacterial infections in excess of 200 ug/ml [31–34]. *In* C57BL/6J mice fed with aluminum sulfate for 5 months CRP was found to increase to ~26.2 ug/ml or ~13.1-fold over ‘0’ time controls consistent with the notion of a mild systemic inflammation in aluminum-sulfate fed C57BL/6J mice versus age-matched, magnesium sulfate-fed controls.

#### Interleukin-6 (IL-6)

The 212 amino acid (23.7 kDa; encoded at chr 7p15.3) pro-inflammatory cytokine interleukin-6 (IL-6), involved in the immune response, hematopoiesis, platelet production and the acute phase reaction is a circulating inflammatory mediator and biomarker. In previous independent studies IL-6 was found to average between ~3 pg/ml to 67 pg/ml in human blood serum and this value is similar to the ~2.5 pg/ml found in C57BL/6J mice in the current investigation [35,36]. Interestingly, c*i*rculating IL-6 levels are known to increase modestly with advancing age and their excessive presence in the blood serum is a risk factor and potential biomarker for various inflammatory diseases including SI and various, seemingly unrelated, inflammation-associated disease states that include AD, systemic bacterial infection, fever, autoimmune disease, type II diabetes and periodontal disease [35–38].

#### Tumor necrosis factor alpha (TNFα)

The 233 amino acid (25.6 kDa; encoded at chr 6p21.33) tumor necrosis factor alpha (TNFα), also known as cachectin, is an O-glycosylated, homotrimeric cytokine involved in the regulation of a wide spectrum of biological processes that include cell proliferation, differentiation, apoptosis, lipid metabolism, coagulation, insulin resistance and cancer. Similar to IL-6, TNFα is a relatively abundant circulating inflammatory mediator-biomarker whose levels are positively correlated to the physiological state of systemic inflammation [1–4,39–43]. Interestingly this pro-inflammatory cytokine may also play a role in neuroprotection and increases in the serum may be part of an inflammatory sensing-and-signaling system linked to physiological attempts at CNS and PNS cell repair and neutralization of SI [40–42]. Average published concentrations of TNFα reported are 4–11 pg/ml in human serum [40–42]. Interestingly we observed that after 5 months of dietary aluminum sulfate exposure there was an increase in C57BL/6J mouse serum of TNFα, an increase that is significant and highly suggestive of the establishment of a systemic inflammation in the murine circulatory system [1–4,42,43].

#### Increases in small non-coding RNA (sncRNA) microRNAs miRNA-9, miRNA-125b and miRNA-146a

The pro-inflammatory microRNAs miRNA-9-1 (encoded in humans at chr 1q22), miRNA-125b (encoded at chr 11q24) and miRNA-146a (encoded at chr 5q33.3): (i) are detectable in human biofluids including extracellular fluid (ECF) and cerebrospinal fluid (CSF) [11,17,20]; (ii) are increased in ECF and CSF to various degrees in AD patients [17,20]; and (iii) are each stress-induced miRNAs that contain binding sites for the pro-inflammatory transcription factor NF-kB (p50/p65 complex) in their gene promoters [11,17,20,44–46]. While miRNA-9 and miRNA-125b displayed modest increases in abundance in the blood serum of C57BL/6J mice after aluminum-sulfate treatment, miRNA-146a exhibited the most significant increase in aluminum-treated animals after 5 months (Figures 2A and 2B). Importantly, other single miRNAs or families of miRNAs may be further involved in establishing a pathogenic, pro-inflammatory signaling program, however in the present studies miRNA-146a was found to be the most up-regulated microRNA in serum after aluminum-sulfate treatment. Up-regulated miRNA-146a has been associated with inflammatory neurodegeneration and altered innate-immune responses in the CSF and brain of AD patients [19,22]. Notable miRNA-146a targets in the blood serum and human CNS include the innate-immune regulatory complement factor H (CFH) with increased CNS miRNA-146a leading to decreased CFH in the same neurological compartments [19,21,22,46]. Notably, miRNA-146a has been found to be significantly elevated in mild cognitive impairment (MCI) compared to age-matched controls, suggesting that miRNA-146a or other related pro-inflammatory miRNAs could be used as part of a panel of novel noninvasive biomarkers for biometal dyshomeostasis, toxic metal accumulation, SI and/or the early clinical diagnosis of AD [19,22,43–47].

## Conclusion

This study is the first to show that the circulating cytokines IL-6 and TNFα, CRP and the pro-inflammatory microRNAs miRNA-9, miRNA-125b and miRNA-146 are elevated in mouse blood serum after ingestion of physiologically realistic amounts of aluminum (as sulfate) in the diet. After 5 months of aluminum-sulfate ingestion by C57BL/6J mice, all 6 biomarkers (CRP, IL-6, TNFα, miRNA-9, miRNA-125b and miRNA-146A) were found to exhibit increases in the blood serum, indicating a significant stimulation of SI biomarkers in aluminum-sulfate fed animals. These focused increases: (i) were found to be progressive and aluminum-induced accumulation of inflammatory biomarkers in the blood serum was age-related over the period of 0–5 months; and (ii) this may be reflective of the degree of development of SI expression programs and ensuing neuropathology. Interestingly, similar elevations of many of these circulating cytokines and related biomarkers are known to increase with advancing age and in various inflammation-associated disease conditions that include SI, systemic bacterial infection, fever, autoimmune disease, type II diabetes, periodontal disease and AD [35–42]. Together these blood serum metrics may be useful, as a group, in SI diagnosis, prognosis and/or predictive of chronic inflammatory neurodegeneration and cell death in the CNS [47]. Studies are currently underway to further determine the neurological-cognition status of aluminum-sulfate fed C57BL/6J mice, the status of other pathological markers in experimental SI, and how these compare to similar disease biomarkers in the blood serum and other body fluids of human patients with AD and comorbidities with an inflammatory and neurodegenerative component.

## Abbreviations

AD: Alzheimer’s Disease
ANOVA: Analysis of Variance
C57BL/6J: Common Strain of Laboratory Mouse
C-reactive protein: An acute phase protein and inflammatory marker
IL-6: Interleukin-6, a pro-inflammatory cytokine
miRNA: microRNA
miRNA-146a: microRNA-146a
SI: Systemic Inflammation
